# Preparation and Properties of Multi-Responsive Liquid Crystalline Poly(urethane-acrylate)s and Its Composite Membranes

**DOI:** 10.3390/polym16131854

**Published:** 2024-06-28

**Authors:** Liming Zhou, Ziwen Wang, Lijun Gao, Hongcheng Yang, Shaoming Fang

**Affiliations:** School of Material and Chemical Engineering, Zhengzhou University of Light Industry, Zhengzhou 450000, China

**Keywords:** azobenzene, liquid crystal polymer, composite membranes, electrospinning, photodeformation

## Abstract

In this work, a kind of side chain liquid crystalline poly(urethane-acrylate)s was synthesized by free polymerization based on self-made liquid crystalline monomers, and a series of liquid crystalline polyurethane/shape memory polyurethane composite membranes were prepared by electrospinning. The synthesized liquid crystalline poly(urethane-acrylate)s have excellent thermal stability. Due to the regular arrangement of azobenzene on the side chains, polymers can rapidly undergo a photoinduced transition from trans-isomerism to cis-isomerism in THF solution and restore reversible configurational changes under visible light. The composite membranes prepared by electrospinning can also undergo photoinduced deformation within 6 s, and the deformation slowly returns under visible light. Meanwhile, the composites have shape memory, and after deformation caused by stretching, the membranes can quickly recover their original shape under thermal stimulation. These results indicate that the composites have triple response performances of photoinduced deformation, photo-, and thermal recovery.

## 1. Introduction

Stimulus-responsive polymers actively produce predetermined geometric changes when stimulated, making it possible to develop polymers with remote drive capabilities [1,2,3]. Among them, the photoresponsive polymer with photomechanical conversion capabilities is a new star in the field of smart materials, because it has the advantage of remote, instantaneous, local, and precise control [4,5,6].

Liquid crystal polymers (LCP), especially ordered liquid crystal polymers, have significant photodeformability and the potential to construct light-driven soft actuators [7]. Liquid crystalline polyurethane (LCPU) is a kind of block copolymer with alternating soft and hard segments resulting from the introduction of urethane bond(-NHCOO-) into the liquid crystalline polymer. LCPU is usually synthesized through condensation polymerization and free radical polymerization [8,9]. One method of preparing LCPU by condensation polymerization is to react isocyanate with polyol, while the other entails the reaction of alcohols with liquid crystal units and isocyanates [10]. The prepolymer obtained by these two methods reacts with chain extenders to obtain LCPU. The preparation of LCPU by free radical polymerization involves the addition of acrylate to liquid crystalline monomers, which have both unsaturated double bonds and urethane bonds, and then the initiation of chain polymerization with an initiator to obtain liquid crystalline poly(urethane-acrylate)s [11]. In terms of structure, according to the relative position of the molecular chain where the liquid crystal unit is located, it can be divided into main chain liquid crystal polyurethane (MLCPU), side chain liquid crystal polyurethane (SLCPU), and composite (main/side chain) liquid crystal polyurethane. This type of material has many application prospects, among which the most notable is the photo responsive polyurethane. Compared with traditional thermoresponsive liquid crystal polymers, the many advantages of photoresponsive polymers contribute to the development of various smart devices that can be operated remotely [12]. Common photosensitive groups include azobenzene (azo), spiropyran (SP), and diarylethene (DTE). Azobenzene and its derivatives, as the most common photoresponsive group, are often added to liquid crystal materials. Under 340–380 nm ultraviolet light irradiation, the azobenzene group is the cis and trans isomerization in the microstructure [13,14]. Compared with other photoresponsive materials, azophenyl liquid crystal polymers (azo-LCP) can form different micro/nanostructures and reversibly adjust the chemical environment and/or morphology [15,16]. Under light irradiation, the azophenyl groups in azo-LCP undergo reversible trans-cis isomerization, resulting in changes in the microchemical properties and interfacial energy of micro/nanostructures [17,18]. In recent years, researchers have reported on a large number of LCPU materials, combining the unique photoresponse of the azobenzene structure with the good mechanical properties of polyurethane (PU) [19,20]. For example, Annapooranan et al. significantly improved the thermal stability and fatigue resistance of azobenzene liquid crystal elastomers by selecting a suitable PU composition [21]. Shen et al. prepared LCPU membranes containing azophenyl groups in the side chain. These membranes have a good thermal- and photoresponsive shape memory ability, which improves the mechanical properties of these polymers [22]. As an advanced intelligent material, LCPU has been widely used in the fields of response stimulus material, functional memory material, and self-healing material. At present, many composite materials have been prepared by blending LCPU with other flexible polymers. Although the photoresponsive properties of the composites are enhanced, the mechanical properties are reduced, and the application of the materials is limited to a certain extent [23].

In this study, based on a new type of liquid crystal monomer with the azobenzene and double bond groups, three types of liquid crystal poly(urethane-acrylate)s were synthesized by free radical polymerization. Then, the liquid crystal polymers were combined with shape memory polyurethane to prepare the composite membranes by electrospinning technology. The effects of the structure and content of the materials on liquid crystal, optical and thermal response properties of poly(urethane-acrylate)s and composite membranes were studied in detail. The prepared composite materials are expected to be applied in the fields of photoresponse controller.

## 2. Materials and Methods

### 2.1. Materials

**Figure 1:**
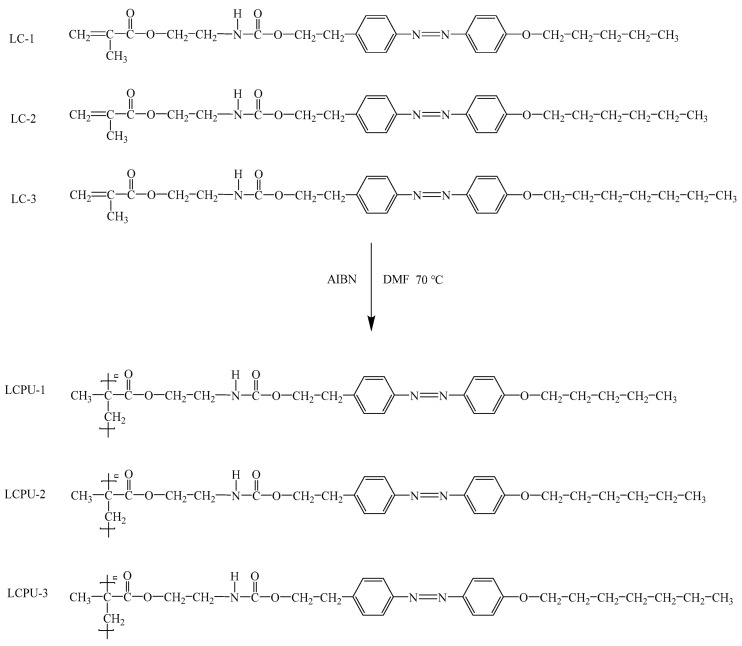
Synthesis route of LCPU-1, LCPU-2, and LCPU-3.

The chemical compounds 2,2′-azobis (2-methylpropionitrile) (AIBN) and isophorone diisocyanate (IPDI) were purchased from Shanghai Mackin Biochemical Co., Ltd. (Shanghai, China). Polycaprolactone diol (PCL-3000) was purchased from Shanghai D&B Biotechnology Co., Ltd. (Shanghai, China). Liquid crystal monomers (LC-1, LC-2, LC-3) (Figure 1) were made in our laboratory, and N,N-dimethylformamide (DMF) and tetrahydrofuran (THF) were purchased from Tianjin Fuyu Fine Chemical Co., Ltd. (Tianjin, China).

### 2.2. Synthesis of Poly(urethane-acrylate)s Containing Azophenyl Groups in Side Chains

The synthetic route of poly(urethane-acrylate)s is shown in Figure 1. A total of 2 g of liquid crystal monomer LC-1 and 1.5% AIBN were dissolved in 8 g of DMF and the reaction was carried out at 70 °C for 24 h. After the solution was cooled to room temperature, the solution was gradually added to ethanol and, finally, the orange filter residue was obtained after filtration. The filter residue was dried in a vacuum drying oven at 80 °C for 24 h to obtain an orange solid, which was liquid crystalline poly(urethane-acrylate) LCPU-1. According to the above experiment, LCPU-2 and LCPU-3 were obtained by replacing LC-1 with LC-2 and LC-2. The yields of LCPU-1, LCPU-2, and LCPU-3 were 82.4%, 79.2%, and 80.8%, respectively.

### 2.3. Synthesis of Shape Memory Polyurethane (SMPU)

First, 60 g of polycaprolactone glycol (average molecular weight: 3000 g/mol), 4.44 g of IPDI, and 1 ‰ of DBTL were added to the flask and stirred for 0.5 h and 20 min at 60 °C and 80 °C, respectively. The temperature was increased to 140 °C at a 20 °C gradient interval and stirred continuously, and then it was put into a 180 °C vacuum drying oven for 8 h. Finally, it was cooled to produce a milky white solid, which was shape memory polyurethane (SMPU).

### 2.4. Preparation of SMPU Fiber Membranes

First, the mixed solvent was prepared by combining tetrahydrofuran (THF) and N, N-dimethylformamide (DMF) in a 1:1 volume ratio. Then, 2 g SMPU was dissolved in 18 g mixed solvent to obtain the spinning solution with a concentration (mass fractions) of 10%. According to a similar method, spinning solutions with concentrations of 12%, 15%, and 18% can be obtained. Taking 10% polyurethane spinning solution as an example, 10 mL of spinning solution was extracted with a needle tube and put into the electrospinning machine. The flow rate of the micropropulsion pump was adjusted to 2 mL/h, the voltage was 13 kV, and the diameter of the needle was 0.6 mm. The 10 mm thick aluminum foil was used to receive the filaments ejected from the needle on the roller receiver, and the rotational speed of the roller receiver was 200 r/min. The above process was repeated to obtain SMPU fiber membranes with different concentrations of spinning solution.

### 2.5. Preparation of Composite Fiber Membranes

The optimal concentration of SMPU electrospinning fiber membrane is 10% and the fiber diameter distribution determined by SEM is the narrowest, so a spinning solution with a mixed concentration of SMPU and poly(urethane-acrylate)s of 10% was prepared. As shown in Table 1, the ratio of SMPU to poly(urethane-acrylate) was 2:1, 4:3, 1:1, and the total mass concentration was 10%.

### 2.6. Characterization

Nuclear magnetic resonance spectroscopy (^1^H-NMR, ^13^C-NMR) were tested by the AVANCEIIInuclear magnetic resonance instrument (Bruker, Germany) with deuterated chloroform (CDCl_3_) at the frequency of 600 MHz. The Fourier transform infrared spectroscopy of Thermo Fisher Scientific (Waltham, MA, USA) (Nicolet iS5) was used to test infrared spectra of materials. The scanning range was 500–4000 cm^−1^, the scanning time was 16, and the scanning mode was attenuated total reflection (ATR). The American TA company (Austin, TX, USA) Q20 differential scanning calorimeter (DSC) was used to measure phase change temperature. The sample mass was 3–5 mg under N_2_ conditions. The temperature range was 25–200 °C, and the heating and cooling rate was 5 °C/min. Phase texture was observed using XPF-500C polarizing microscope (POM) of Shanghai Zekang Optics (Shanghai, China). The small number of samples were first placed on a slide with a heating table and the sample temperature was changed to observe the liquid crystal texture. A Bruker D8 Advance X-ray powder diffractometer was used for the x-ray diffraction (XRD) experiments, using a Cu Kα x-ray tube with a wavelength of λ = 1.5406 Å, running at 40 kV and 40 mA. The range of 2theta was 10–35°, the step size was 0.05°, and the residence time was 0.8 s. The temperature during testing was 90 °C, 120 °C, and 150 °C. A diamond TG/DTA comprehensive thermogravimetric analyzer from Perkin Elmin (Waltham, MA, USA) was adopted to characterize the thermal stability of materials. The sample mass was 3–5 mg, N_2_ protection was used, the scanning range was 25–800 °C, and the heating rate was 10 °C/min. UV–vis absorption spectra were measured using a spectrometer (Hitachi U-3900H, Tokyo, Japan) (Hitachi U-3900H, Tokyo, Japan). The sample concentration was 27 mg/L (THF as solvent), the scanning range was 250–800 nm, and the scanning rate was 300 nm/min. The morphology of the materials was assessed by field emission scanning electron microscope (SEM, JSM-7001F, JEOL Ltd. Tokyo, Japan). The polymer membranes were stuck on the object table with conductive adhesive, sprayed with gold for 180 s, sent to the instrument, and the scanning voltage was 15 kV. A UV–LED spot curing system (HTLD-4 II) was used to test photoisomerization and the photoinduced deformation performances. The wavelength and power of the UV lamp were 365 nm and 3 W, respectively, and the distance between the light source and the fiber membrane was 2 cm. The area of the facula was 2.25 cm^2^; therefore, the light intensity was 1.33 W/cm^2^.

## 3. Results and Discussion

### 3.1. Structural Analysis of Poly(urethane-acrylate)s

**Figure 2:**
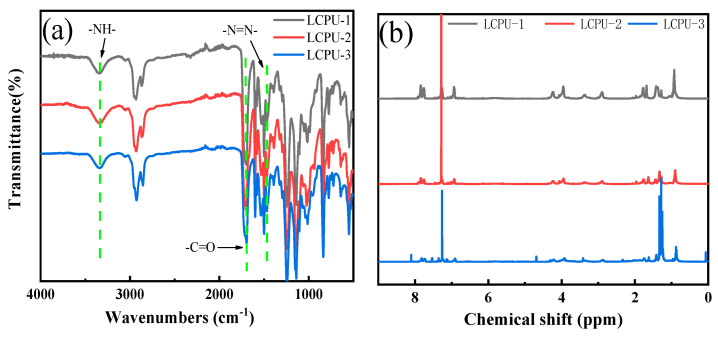
Infrared spectra (**a**) and ^1^H−NMR spectra (**b**) of LCPU-1, LCPU-2, and LCPU-3.

The infrared spectra of poly(urethane-acrylate)s are shown in Figure 2a. Based on the infrared spectra, the stretching vibration absorption peak of -N=N- in the azophenyl group at 1460 cm^−1^ is obvious. The stretching vibration absorption peak of -NH at 3300 cm^−1^ and the strong absorption peak of C=O at 1700 cm^−1^ fully proved the existence of the -NHCOO- group. At the same time, the peak of -C=C- at 1630 cm^−1^ disappeared. The above results prove the existence of side chains in polymers, and the free radical polymerization of these liquid crystal monomers was generated in solution, verifying the successful synthesis of poly(urethane-acrylate)s.

Figure 2b is the ^1^H-NMR spectra of LCPU-1, LCPU-2, and LCPU-3. It can be seen that chemical shifts of hydrogens (=CH_2_) on the double bond in methacrylate at 5.6 ppm and 6.1 ppm disappeared obviously. The results show that liquid crystal monomers were polymerized and poly(urethane-acrylate)s were synthesized successfully.

### 3.2. Liquid Crystal Properties of Poly(urethane-acrylate)s

**Figure 3:**
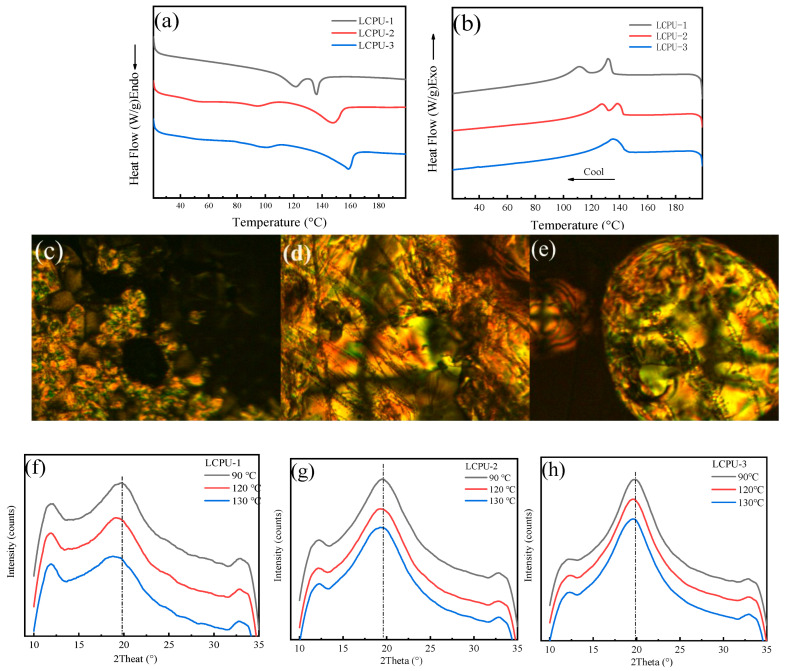
DSC curves (**a**,**b**), POM images (**c**–**e**), and XRD patterns (**f**–**h**) of LCPU-1, LCPU-2, and LCPU-3. (**a**) Heating curves, (**b**) cooling curves, (**c**) LCPU-1, (**d**) LCPU-2, (**e**) LCPU-3, temperature: 130 °C, ×100, (**f**) LCPU-1, (**g**) LCPU-2, (**h**) LCPU-3.

The liquid crystal properties of poly(urethane-acrylate)s were studied by DSC and POM. Figure 3a,b show the DSC curves during the heating and cooling processes, respectively. The three types of polymers exhibit two endothermic peaks in Figure 3a. The phase transition temperature of the first endothermic peak from solid state to liquid crystal state was the melting temperature (T_m_), and the T_m_ of LCPU-1, LCPU-2, and LCPU-3 were 120 °C, 95 °C, and 100 °C, respectively. The second endothermic peak from liquid crystal state to liquid state corresponded to the clear point temperature (T_i_) of the LCPUs, and T_i_ was 136 °C (LCPU-1), 148 °C (LCPU-2), and 158 °C (LCPU-3). It can be seen from Figure 3a that the liquid crystal temperature range of polymers gradually widened with the increase in the number of alkyl chains in the side chain, and T_m_ gradually decreased, but T_i_ gradually increased. The reason for this change is mainly related to the van der Waals forces and the spatial arrangement among molecules. The longer the molecular chain of liquid crystal molecules, the greater the distance separating molecules. Also, the lower the van der Waals forces, the lower the T_m_, and the longer the alkyl chain, the looser the spatial arrangement of molecules and, therefore, the easier the formation of the liquid crystal phase. Therefore, its T_i_ increased, causing the isotropic points to become higher and higher.

Figure 3c–e show the POM images of LCPUs. When the temperature rose to the clear spot temperature, the visual field appeared to be dark, the phenomenon of birefringence was not observed, and LCPUs were in liquid state. When the temperature was lowered to 130 °C (a temperature between T_m_ and T_i_) and maintained for 10 min, the birefringence phenomenon of LCPUs obviously occurred, indicating that polymers were in the liquid crystal state. However, the textures of all the polymers were not observed clearly. This phenomenon may be due to the high viscosity of the polymer caused by hydrogen bonds between the molecular chains which may hinder the formation of well-defined liquid crystal textures [24,25].

The XRD patterns of the materials are shown in Figure 3f–h. It was found that the three types of polymers exhibited significant X-ray diffraction peaks at 120 °C and 150 °C (which are within the liquid crystal range of polyurethane). However, compared to the diffraction peak at 90 °C (which is lower than the melting temperature), the peak position was shifted, moving toward a lower angle [26,27]. This further confirms that the three liquid crystal polymers undergo a phase transition within the liquid crystal range.

### 3.3. Thermal Stability of Poly(urethane-acrylate)s

**Figure 4:**
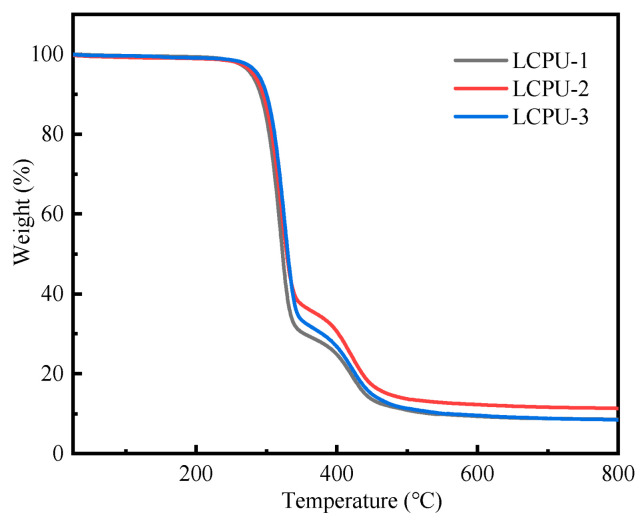
TG curves of LCPU-1, LCPU-2, and LCPU-3.

It can be seen from Figure 4 that the TG curves of poly(urethane-acrylate)s were similar. The three polymers hardly decomposed before 250 °C and degraded completely around 500 °C, indicating that the materials had good stability. In the TG curves, the degradation temperature of the first stage was around 280 °C, caused by the fracture of the amino ester bond (-NHCOO-). The degradation temperature of the second stage was around 380 °C, mainly as a result to the decomposition of -C-C-. Because the LCPUs had similar structures, their TG curves were similar.

### 3.4. The Photoresponse Performances of Poly(urethane-acrylate)s

**Figure 5:**
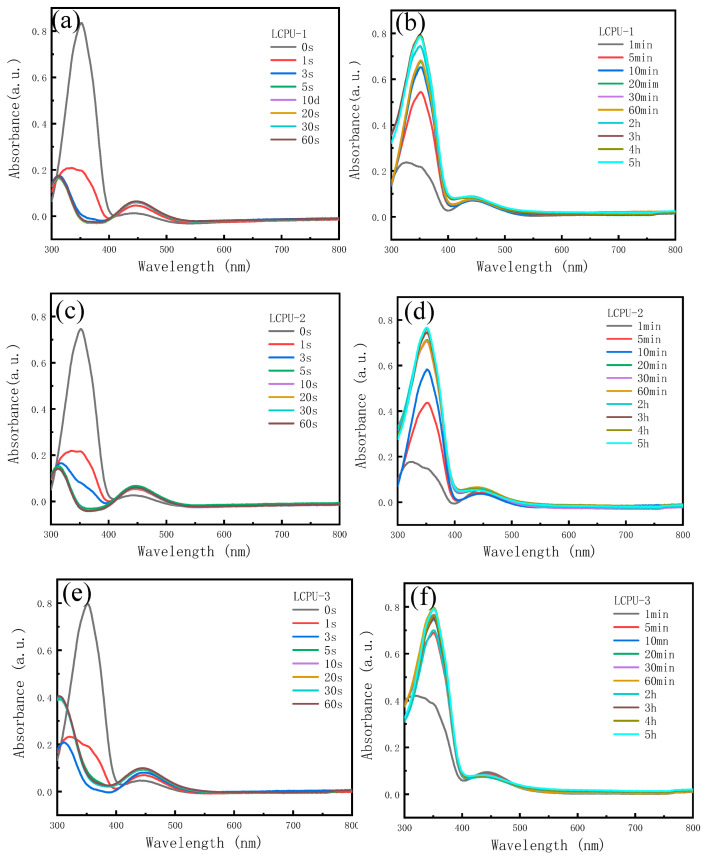
UV–vis absorption spectra of LCPU-1, LCPU-2, and LCPU-3 solution. (**a**,**c**,**e**): different irradiation times under 365 nm ultraviolet light; (**b**,**d**,**f**): different placement times under visible light.

The UV absorption intensity of poly(urethane-acrylate)s solution was determined at different 365 nm UV light irradiation times (the area of the facula was 2.25 cm^2^ and the light intensity was 1.33 W/cm^2^) to study the photoresponsivity of polymers. The results are shown in Figure 5. The left side is UV–vis spectral changes dependent on time of LCPU-1, LCPU-2, and LCPU-3 solution upon irradiation with 365 nm UV light. and the right side is the restored spectra under visible light for different times. The absorption peak of the azobenzene group in trans configuration occurred at 350 nm, and that of the cis configuration occurred at 450 nm. When polymers were irradiated by 365 nm ultraviolet light, the absorption peak at 350 nm gradually weakened, and the absorption peak at 450 nm gradually increased. The azobenzene group gradually changed from a trans configuration to a cis configuration. With the increase in alkyl chain length in the side chain, the photoresponse speed gradually decreased, showing a trend where LCPU-1 was faster than LCPU-2 and LCPU-3. It can be seen that LCPU-1, with the fastest photoresponse speed, realized the change from a trans configuration to a cis configuration of the azobenzene group within 5 s. During the restoration process under visible light, LCPU-3, with a long alkyl chain, exhibited a significantly faster recovery rate than that associated with the other two kinds of liquid crystal polyurethane. Moreover, polyurethanes were basically able to recover from cis-to-trans configuration within 5 h under visible light.

### 3.5. Microstructure of SMPU Fiber Membranes

**Figure 6:**
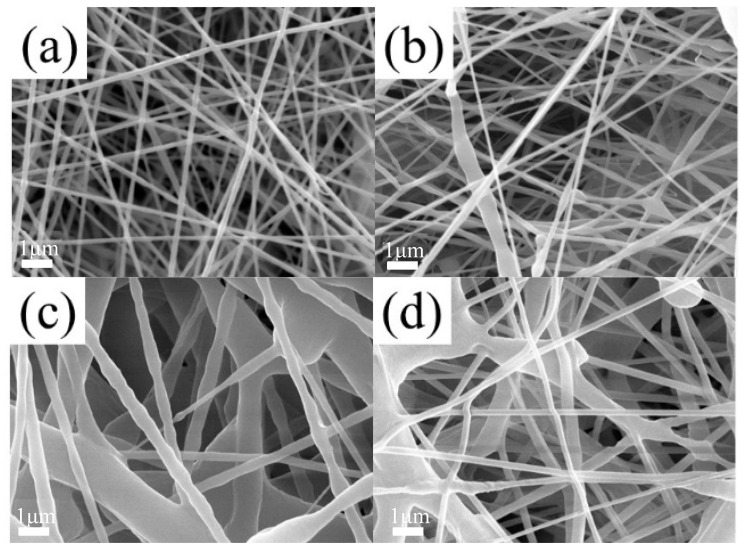
SEM images of SMPU fiber membranes. (**a**) Concentration of 10%, (**b**) concentration of 12%, (**c**) concentration of 15%, and (**d**) concentration of 18%.

Different fiber membranes were prepared by electrospinning, and their SEM images are shown in Figure 6. In Figure 6b–d, obvious string of beads can be observed. The diameter of the fibers shown in Figure 6c,d was uneven, and the diameter of the widest part could reach 1 μm. Figure 6a shows the fiber membranes prepared by spinning solution (concentration of 10%). The diameter of the fiber was uniform, with an average diameter of about 100 nm, without obvious beading and fracture defects. To sum up, the most appropriate concentration with the most uniform fiber diameter and no obvious defects was 10%. With the gradual increase in the concentration, the fiber diameter distribution gradually widened and obvious beading and adhesion defects appeared. This is because, under the set voltage and propulsion speed, the spinning solution with a higher concentration adhered more easily after being ejected from the pinhole, so more defects were generated. In conclusion, the concentration selected for the preparation of composite membranes was 10%.

### 3.6. Microstructure of Composite Fiber Membranes

**Figure 7:**
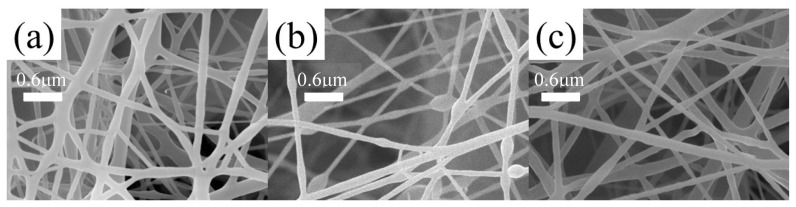
SEM images of LCPU-1/SMPU composite fiber membrane, (**a**) ratio of 1:2, (**b**) ratio of 3:4, (**c**) ratio of 1:1. The total mass concentration is 10%.

**Figure 8:**
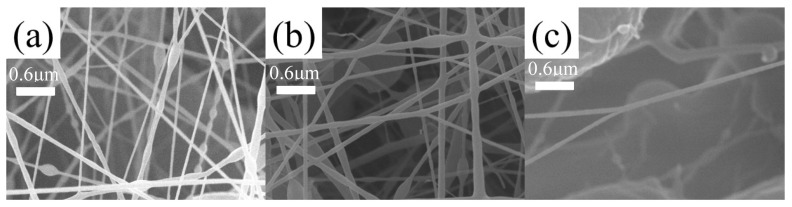
SEM images of LCPU-2/SMPU composite fiber membranes, (**a**) ratio of 1:2, (**b**) ratio of 3:4, (**c**) ratio of 1:1. The total mass concentration is 10%.

**Figure 9:**
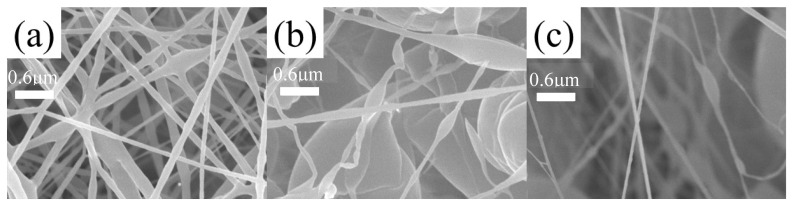
SEM images of LCPU-3/SMPU composite fiber membranes, (**a**) ratio of 1:2, (**b**) ratio of 3:4, (**c**) ratio of 1:1. The total mass concentration is 10%.

The micro morphology of the composite fiber membranes prepared by electrospinning is shown in Figure 7, Figure 8 and Figure 9. In Figure 7a, there are obvious defects and an uneven fiber thickness. In Figure 7b, there are visible beading and fracture defects, but the fiber diameter is uniform, with an average diameter of 100 nm. In Figure 7c, the fiber has no obvious defects.

In Figure 8a, the single fiber has obvious beading defects and is not uniform. The difference in the diameter of the fibers is obvious. Within the observation range, the maximum diameter was 100 nm, and the minimum diameter was close to 50 nm. In Figure 8b, the mass ratio of LCPU-2 and SMPU is 3:4 and the defects of the fibers are more obvious with fractures and larger beading phenomena, but the diameter of the fibers is more uniform and the diameter difference is smaller. In Figure 8c, the composite material has poor silk forming effect and obvious adhesion. To sum up, in the LCPU-2/SMPU system, the effect of LCPU-2 and SMPU with a mass ratio of 3:4 was superior to the effect of the other two systems, but these three proportions of fiber membranes all had obvious defects. In this system, where SMPU was the framework support, the effect was more obvious. With the increase in LCPU-2 content, the viscosity of the spinning solution gradually decreased, so the defect became more obvious.

In Figure 9a, obvious filaments can be observed, but there is adhesion. The diameter is relatively uniform compared with that associated with the other two proportions. The maximum diameter was about 170 nm and the minimum was about 90 nm. In Figure 9b,c, the phenomenon of adhesion and beading is more obvious, and even the fiber in Figure 9b exhibits the phenomenon of curling, which may be caused by too many and large beading. In Figure 9c, there are obvious filaments with a diameter of about 100 nm, but there are also bead strings and even branching. The above situation may be related to the increase in LCPU-3 content.

It can be seen that the length of the side chains in poly(urethane-acrylate)s had a significant impact on electrospinning. The longer the side chains, the easier the molecular chains became entangled with each other, resulting in a higher system viscosity. Therefore, LCPU-1/SMPU can be spun at a high mass ratio, but LCPU-3/SMPU can only be spun at low content.

### 3.7. Photoresponse Behavior of Composite Fiber Membranes

**Figure 10:**
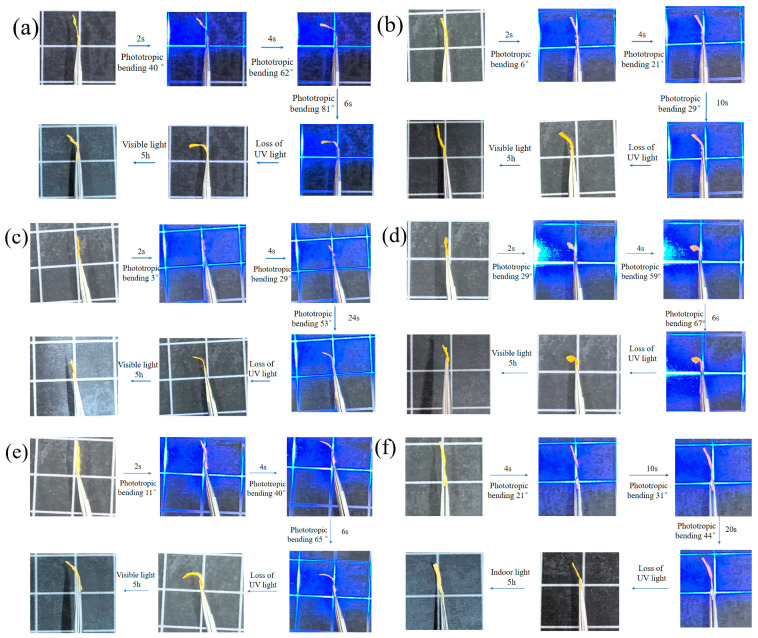
Pictures of the photoresponse process of composite electrospinning membranes, (**a**) LCPU-1:SMPU = 1:2, (**b**) LCPU-1: SMPU = 3:4, (**c**) LCPU-1:SMPU = 1:1, (**d**) LCPU-2: SMPU = 1:2, (**e**) LCPU-3: SMPU = 1:2, (**f**) LCPU-3: SMPU = 3:4.

The photodeformation and visible light recovery behavior of the composite membranes is shown in Figure 10. In all the membranes, when LCPU-1:SMPU = 1:2, the UV light response speed of the composite membranes was the fastest, the change from trans to cis azobenzene groups was basically completed in about 6 s, and the composite membranes completed the bending, that is, the liquid crystal polymer with shorter side chains had a faster UV light response speed. With the increase in azobenzene content, the UV response time of the composite membrane increased, a phenomenon which may be due to the fact that a greater content of azobenzene groups would require more energy and a longer illumination time. The same phenomenon also existed in the LCPU-2/SMPU and LCPU-3/SMPU systems. In addition, the quality of the fiber had a significant impact on the light response speed. In the LCPU-3/SMPU system, when the content of LCPU-3 was high, the electrospinning fibers were uneven, resulting in a slower light response.

### 3.8. Thermal Response of Composite Membranes

**Figure 11:**
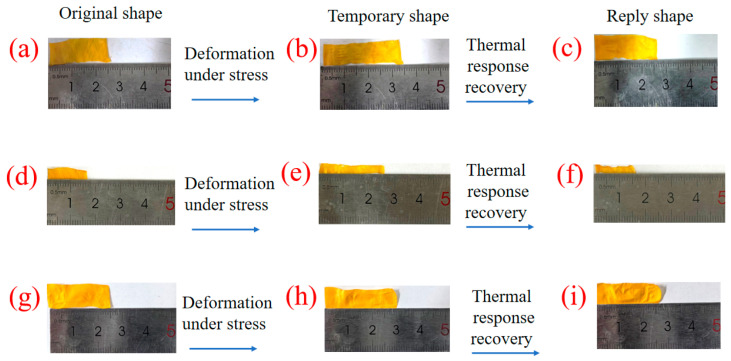
Pictures of the thermal response process of the composite membranes, (**a**–**c**) LCPU-1:SMPU = 1:2, (**d**–**f**) LCPU-1:SMPU = 3:4, (**g**–**i**) LCPU-1:SMPU = 1:1.

Figure 11 shows the tensile deformation and thermal response recovery behavior of the composite membranes. The composite membranes prepared using the LCPU-1/SMPU system were stretched and deformed and then put into the 70 °C oven. The shape recovery of the composite membranes was achieved in about 5 s, verifying the thermal response recovery ability of the composites. According to the calculated data in Table 2, it can be concluded that the thermal response recovery rate of the composite membranes exhibited a significant downward trend with the increase in LCPU-1 content. With the increase in LCPU-1 content, the content of shape memory polyurethane decreased, and the synergistic effect between LCPU-1 and the shape memory polyurethane decreased, resulting in a decrease in the recovery rate of the composite material.

Membranes transformed the microscale change in the length of azobenzene molecules into the macroscopic bending behavior due to the photoresponse, resulting in photoinduced bending. Subsequently, under heating or visible light conditions, the cis isomer could be reversibly transformed into the trans isomer and the shape of the composite membrane could be restored. Through the alternating action of light and heat, the composite membranes were able to undergo photoinduced deformation or motion.

Compared with other photoresponsive polymers [28,29,30,31] (Table 3), LCPU-1/SMPU has a faster light response speed and is associated with a better shape memory performance. Therefore, the composite fiber membranes may be applied in the field of optical response drivers.

## 4. Conclusions

In summary, the synthesized poly(urethane-acrylate)s with azophenyl groups are side chain liquid crystal polymers with good thermal stability, and the liquid crystal range of the polymer widens with the increase in alkyl chain length in the side chains. The liquid crystal polymers exhibit a rapid response to 365 nm ultraviolet light in THF solution. LCPU-1 completes the response to 365 nm UV light in about 5 s and realizes the configuration change of the azobenzene group from trans to cis. In addition, under visible light, cis isomerism can gradually return to the trans isomerism of azobenzene. Among the three polymers, the length of the side chain has a significant effect on the photoresponse speed. The shorter the side chain, the faster the light response speed. The length of the side chain has a significant impact on electrospinning. LCPU-1/SMPU with a short side chain length can be spun at a high mass ratio and the diameter of the fibers remains uniform. When the mass ratio of LCPU-1 and SMPU is 1:2, the composite membranes can realize photoinduced bending within 6 s. Meanwhile, the membranes can achieve thermal response recovery after tensile deformation, with a shape recovery rate exceeding 88%. These results prove that the obtained composite membranes have a fast UV light response behavior and photo/thermal recovery. Based on the fast response speed of the composite membranes and the good biocompatibility of PCL, the composite materials have application potential in biomedicine, photoresponse driver, and other applications.

## Figures and Tables

**Table 1:** The mass proportion of poly(urethane-acrylate)s and SMPU preparation solutions.

LCPU-1:SMPU	LCPU-2:SMPU	LCPU-3:SMPU
1:2	1:2	1:2
3:4	3:4	3:4
1:1	1:1	1:1

**Table 2:** Thermal response recovery of composite membranes.

Mass Ratio	Tensile Deformation Rate (%)	Recovery Rate (%)
LCPU-1:SMPU = 1:2	36.0	99.6
LCPU-1:SMPU = 3:4	62.5	94.1
LCPU-1:SMPU = 1:1	20.0	88.0

**Table 3:** Performances of LCPU-1/SMPU and other materials reported in previous literature.

Sample	Photoisomerization		Photoinduced Bending			Shape Recovery Rate (%)	Ref.
	λ (nm)	t (s)	λ (nm)	t (s)	Angle (°)		
LCPU-1/SMPU	365	5	365	6	81.0	99.6	This work
MLCPU	365	8	365	5	7.0	85.1	[28]
HAzo-LCEs	365	10	365	10	48.0	-	[29]
SCLCPU(AZO)	450	30		-	-	95.3	[30]
LCPUE	365	285	365	300	13.0	-	[31]

## Data Availability

The original contributions presented in the study are included in the article, further inquiries can be directed to the corresponding author/s.
